# Identification of Chemical Constituents in the Extract and Rat Serum from Ziziphus Jujuba Mill by HPLC-PDA-ESI-MSn 

**Published:** 2014

**Authors:** Shuping Wang, Jingze Zhang, Zhidan Zhang, Wenyuan Gao, Yanan Yan, Xia Li, Changxiao Liu

**Affiliations:** a *Department of Pharmacy, Tianjin Provincial Corps Hospital, Chinese People’s Armed Police Forces, Tianjin 300162 *.; b *Department of Pharmacy, Logistics College of Chinese People’s Armed Police Forces, Tianjin 300162, China .*; c *School of Pharmaceutical Science and Technology, Tianjin University, Tianjin 300072, China .*; d *The State Key Laboratories of Pharmacodynamics and Pharmacokinetics, Tianjin, 300193, China. *

**Keywords:** *Ziziphus jujuba* Mill, Chemical constituents, Metabolites in serum, HPLC-PDA-ESI-MSn, Quantitative analysis

## Abstract

Chinese jujube (*Ziziphus jujuba *Mill.) has long been widely used for human consumption and medicinal purposes in China. It has been reported to possess several vital biological activities. However, the systematic study on the chemical constituents absorbed into plasma and their metabolites is still insufficient.A high-performance liquid chromatography-photodiode array detector-electrospray ionization ion-mass spectrometry (HPLC-PDA-ESI-MS^n^) method was established to analyze the ethanol extract in *Ziziphus jujuba *Mill and the constituents absorbed into rat serum. In the present study, a dose of 10 mL/Kg of ethanol extract of jujube, which is equivalent to 12.5 g crude dried herb/Kg, was orally administrated to rats. The main components were analyzed in the ethanol extract of *Ziziphus jujuba *Mill and the parent constituents and metabolites were studied in rat plasma samples after oral administration of the ethanol extract of jujube.D101 macroporous polystyrene resin was a good pretreatment method to obtain better separation and impurity removal effect. Twenty-two compounds were identified in the ethanol extract of *Ziziphus jujuba *Mill. Four parent compounds and four metabolites were detected in rat serum. Among them, seventeen compounds were reported for the first time.

## Introduction

Chinese jujube, an edible and medicinal fruit, is officially listed in Chinese Pharmacopoeia (1) and Japanese Pharmacopoeia (2). The official jujube, Dazao in Chinese, is prescribed as the dried fruit of *Ziziphus jujuba *Mill. And it is indigenous to China with a history of over 4000 years which belonging to the sect Ziziphus Mill. of the family Rhamnaceae. Several types of components including triterpenic acids, flavonoids, cerebrosides, amino acids, phenolic acids, microelements, vitamins, total sugars and nucleosides have been isolated and identified during the past decades (3, 4). Modern pharmacological studies have shown that Dazao is commonly used for tranquilizing mind, enhancing the immunologic functions, inhibiting tumor growth, resisting fatigue, beautifying and nourishing face, resisting oxidation and aging, *etc *(5-7). In addition, Chinese jujube has been widely utilized as a food additive and exploited to Chinese beverages and other functional food.

Liquid chromatography coupled with mass spectrometry (LC/MS) is a powerful technique for the analysis of complex botanical extracts (8, 9). MS provides abundant information for structural elucidation of the compounds when tandem mass spectrometry is applied. Electrospray ionization (ESI) is a preferred source due to its high ionization efficiency for broad chemical structures, such as phenolics and flavonoids compounds. When a pure standard is unavailable, a rapid and accurate identification of chemical compounds in medicinal herbs are facilitated by HPLC-ESI-MS^n^. In this study, a systematical analysis of chemical constituents in rat serum after oral administration of the methanol extract Chinese jujube was conducted by HPLC-PDA-ESI-MS^n^ for explaining the effective components *in-vivo *and *in-vitro *of DaZao.

## Experimental

*Chemicals and reagents*

D101 macroporous polystyrene resin was supplied by Dajun Technology Development Co., *Ltd*. (Tianjin, China). L-ascorbic acid and cAMP were purchased form Sigma-Aldrich Inc. (St. Louis, MO, USA). Glucose, Rutin, Quercetin. Oleanolic acid, Gallic acid and Jujuboside A were purchased form the Control of Pharmaceutical and Biological Products (Beijing, China). HPLC grade methanol was from Merck (Darmstadt, Germany). Formic acid was purchased from Tianjin Guangfu Fine Chemical Research Institute (Tianjin, China). Reverse osmosis Milli-Q water (18.2 MΩ) from Millipore (Billerica, USA) was used for all solutions. 

*Instrumentation*

Chromatographic analysis was conducted by an Agilent HPLC system (Agilent Technologies, USA) equipped with a quaternary pump, micro degasser, an auto plate-sampler, a thermostatically controlled column compartment, a photodiode array detector (PDA) and an electrospray ionization ion source (ESI). Mass spectrometry detection was conducted by an Agilent HPLC-MS system containing of a surveyor auto-sampling system (Agilent Technologies, USA), and a LC/MSD Trap XCT electrospray ion trap mass spectrometer.

*Sample preparation*

Chinese jujube, the mature fruit of *Ziziphus jujuba *Mill, was collected from Hebei Province in China, and authenticated by Professor Wenyuan Gao (School of Pharmaceutical Science and Technology, Tianjin University, Tianjin, China). 

The Chinese jujube was air dried in the sun immediately after collection. After that, the samples were stoned and carefully cut into slices. The small cut slices were air-dried at room temperature and ground into powder with a particle size of 80 meshes for further analysis. 

The dried powder (10 g) of *Ziziphus jujuba *Mill. was weighed and extracted with 100 mL 70% ethanol for 1 h in an ultrasonic bath with a frequency of 100 kHz at room temperature, respectively. The solution was then filtered and the supernatant obtained. The extraction procedure was performed three times, and the combined supernatants were concentrated to dryness in a rotary evaporator set to 40 °C. The samples were re-dissolved in ultrapure water in 25 mL brown volumetric flasks and filtered through 0.22 μm microporous membrane for further analysis. 

The ethanol extract solution (25 mL) with a jujube concentration of 0.4 mg/mL was pumped through each glass column (2 cm × 40 cm) wet-packed with D101 resins (25 g on dry basis), at 25 °C. The packed height was 25 cm and the bed volume (BV) of the resin was 50 mL. Following adsorption, the target compounds were successively desorbed by different concentrations of ethanol (0%, 10%, 30%, 50%, 70%, 90% and 95%) at a flow of 1 BV/h. The elution volume of each concentration was kept constant at 2.5 BV. Concentrations of 30%, 50% and 70% eluent in desorption solution were determined by HPLC-PDA-ESI-MS^n^. 

*Animal and serum sample collection*

Twelve male SD rats (body weight, 250 ± 20 g) were divided into a blank group and drug group. For rats of the drug group, a dose of 10 mL/Kg of ethanol extract of jujube, which is equivalent to 12.5 g crude dried herb/Kg, was dissolved in 0.9% sodium chloride solution and administered orally twice a day for five days. Equal dose of 0.9% sodium chloride solution was given to the rats of the blank group. At 1 h after the last administration, a blood sample (5 mL) was collected from each rat in clean glass tubes by puncture of the retro-orbital sinus. The blood was centrifuged at 2500 ×g for 20 min to separate serum, and then the serum of each group was mixed together as the sampling schedule and stored at -20 °C before analysis.

*Bio-Samples preparation*

To release the protein-binding medicinal composition, 50 μL of 20% acetic acid was added into 2 mL serum sample and mixed by vortex, followed by 8 mL methanol added and vortexed again. Then, the solution was centrifuged at 2500×g for 15 min. The supernatant was evaporated to dryness at 40 °C in vacuum, and the residue dissolved in 0.5 mL of 70% methanol. After centrifugation at 6600 ×g for 20 min, 20 μL of the supernatant was introduced into the HPLC system for HPLC-PDA-MS^n^ analysis. The blank serum sample was processed with the same methods as described above. All samples were stored at -20 °C until analysis.

*Chromatography*

LC separation was carried out using a mobile phase consisting of methanol (solvent A) and 0.1% formic acid aqueous solution (solvent B) with a gradient elution procedure as 0-10 min, 40-50% A and 60-50% B; 10-15 min, 50-60% A and 50-40% B; 15-25 min, 60-70% A and 40-30% B; 25-35 min, 70-90% A and 30-10% B. The mobile phase flow rate was 1 mL/min with the injection volume of 10 μL; the column was maintained at 30 °C. The scan range was 190-400 nm, and the monitoring UV wavelength was set at 254 nm.

*Mass spectrometry*

Source settings used for the ionization were: nebulizer gas flow, 70.00 *psi*; dry gas flow, 11.00 L min^-1^; electrospray voltage of the ion source, 3000 V; capillary temperature, 350 °C; capillary exit, 158.5 V; skimmer, 40 V. Nitrogen (>99.99%) and He (>99.99%) were utilized as sheath and lamping gas, respectively. The full scan of ions ranging from m/z 100 to 1000 in the positive and negative ion mode was carried out. The fragment ions were obtained using collision energy of 35% for both MS^2^ and MS^3^ experiments. Analyses were conducted at ambient temperature and the data were operated on the Xcalibur software. 

*Quantitative analysis*

The content of oleanolic acid was determined by HPLC. The HPLC separation was performed on HiQ C18 (4.6 × 250 nm, 5 μm) column with a mixture methanol and water (90:10) as the mobile phase, at the flow rate 1.0 mL/min and the peak was monitored at 210 nm. The content of cAMP was determinated by HPLC. The HiQ C18 (4.6 × 250 nm, 5 μm) column was used as stationary phase; the mobile phase was methanol to phosphoric buffer (20mmol KH_2_PO_4_) (10:90) at 30 °C. The flow rate was 0.6 mL/min and the peak was monitored at 254 nm. Total content of sugars were determined by the enthrone-sulphuric acid method (10). Total content of phenolics were determined according to the Folin-Ciocalteu procedure (11) with little modifications. Flavones were determined according to the methods of Miliauskas (12) with some modification. Flavonols were determined according to Kosalec’s method (13). Total saponins were reacted with vanillin-acetic acid solution and perchloric acid, and then the absorbance of the blank solution was read at 550 nm against the blank (14). 

## Results and Discussion

*Optimum conditions for HPLC-PDA-ESI-MS* ^n^ *analysis*

The solvent system was investigated to ensure lower pressure, greater baseline stability, better resolution and higher ionization efficiency. Methanol, acetonitrile and a series of concentrations of aqueous formic acid solution were prepared for analysis. The best result was achieved when the mobile phase consisted of methanol and 0.1% formic acid aqueous solution. Both positive and negative modes were investigated, and the results showed that they were both sensitive for extract samples analysis. 

However, positive ion mode was more sensitive and could provide more information for serum samples analysis. 

Mass spectrometry analysis of reference compounds.

**Figure 1:**
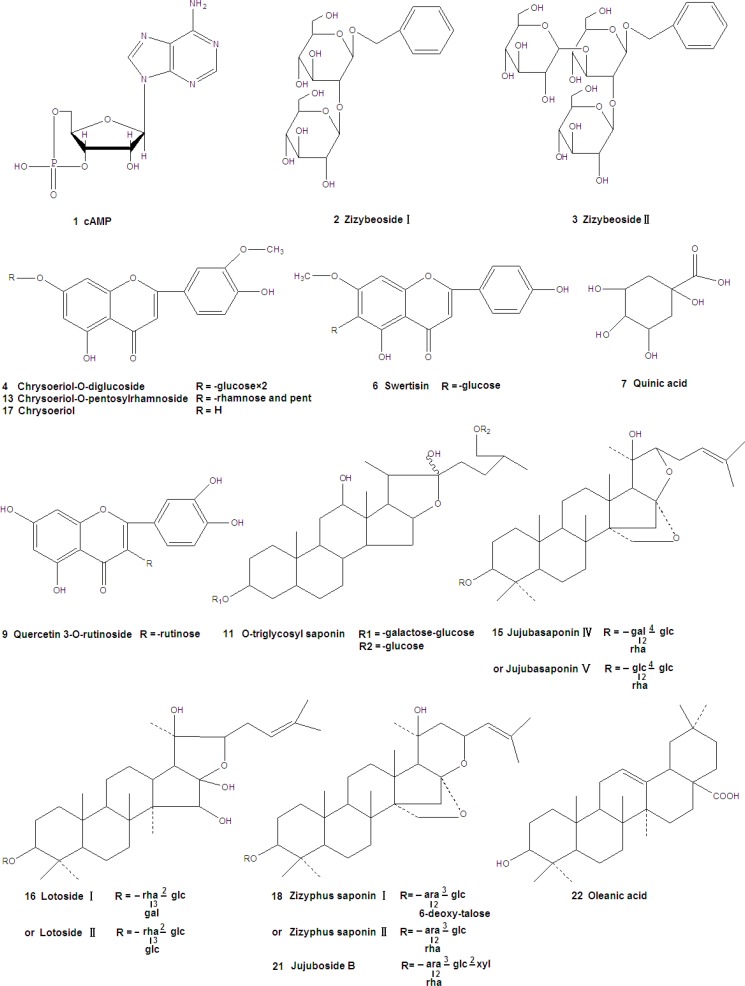
Structures of compounds identified in the extract of Ziziphus jujuba Mill

The structural formulas of standard substances were shown in Figure 1 and the mass spectra were summarized in Table 1. The compounds of different types showed different fragmentation mechanisms. cAMP gave a [M+H]^+^ ion at *m/z *330, and the major product ions were [M+Na]^+^ (*m/z *352), [M+H-194]^+^ (*m/z *136). MS^2^ spectrum of Swertisin (molecular ion at *m/z *[M+Na]^+^ 469) gave characteristic fragment ions of [M+Na-18]^+^ at *m/z *451, [M+Na-120]^+^ at *m/z *349 and [M+Na-120-60]^+^ at *m/z *289. Similar observations were also made by other authors. The pseudomolecular [M+Na]^+^ ion of Jujuboside B was *m/z *1067. Other ion peaks, [M+Na-132]^+^ (*m/z *935) and [M+Na-132-146]^+^ (*m/z *789), were observed. Ions at *m/z *935 and 789 were attributed to the loss of a terminal pentose residue and a rhamnose residue, respectively. Similar observations were also made by Zhao *et al. *(15). Oleanic acid produced a [M+H]^+^ at *m/z *457. Its MS^2^ and MS^3^ spectra gave ions at 439, 393 in positive mode. It was similar to Guo’s study (16).

**Table 1:** Maximum wavelength in UV spectra (λmax) and *m/z *values of ions of standard compounds.

**Standard compounds **	**Molecular formula **	**UV, λ**_max_	-**[M+H]**^+^ **/ [M-**H]	**MS/MS fragments **
cAMP	C_10_H_12_N_5_O_6_P	253	-/330	352,136
Swertisin	C_22_H_22_O_10_	278 ,254	-/469	349,289,203
Jujuboside B	C_52_H_84_O_21_	203	-/1067	935,789
Oleanic acid	C_30_H_48_O_3_	253	-/457	439,393

Six compounds were found in 30% eluent. According to the reference compounds, compound 1 and 6 corresponded to cAMP and Swertisin, respectively. MS^2^ spectra of compound 2 (molecular ion at *m/z *[M-H]^-^ 431) gave characteristic fragment ions of [2M-H]^-^ at *m/z *863, [M-H-162]^-^ at *m/z *269 and [M-H-162-108]^-^ at *m/z *161. [M-H-162]^-^ ion suggested the presence of a hexose. Fragment ion at *m/z *161 suggested the presence of another hexose. Thus, compound 2 corresponded to Zizybeoside Ӏ. The fragmentation mechanisms of compound 3 and 2 were same**. **MS^2^ spectra of compound 3 (molecular ion at *m/z *[M-H]^-^ 593) gave characteristic fragment ions of [M-H-162]^-^ at *m/z *431, [M-H-162-162]^-^ at *m/z *269 and [M-H-162-162-108]^-^ at *m/z *161. It corresponded to Zizybeoside ӀӀ. Zizybeoside Ӏ and ӀӀ were parts of glucosides and they were firstly identified by HPLC-PDA-MS^n^. Their fragmentation mechanisms were summarized. Compound 4 gave a [M-H]^-^ ion at *m/z *623, and the major product ions were [M-H-90]^-^ (*m/z *533), [M-H-162]^-^ (*m/z *461). [M-H-162]^-^ ion suggested the presence of a hexose. According to the MS spectrum and references (Bao *et al., *2009), compound 4 was tentatively indentified as Chrysoeriol-O-diglucoside. Compound 5 generated a [M+Na]^+^ at m/z 766 in MS spectrum and a [M+Na]^+^ at m/z 748([M+Na-18]^+^, lose of H_2_O), 574([M+H-18-132]^+^, lose of pentose), 353, 291(353-162, lose of a hexose) in MS^2^. Thus, compound 5 was presumed O-triglycosyl flavone. 

**Figure 2:**
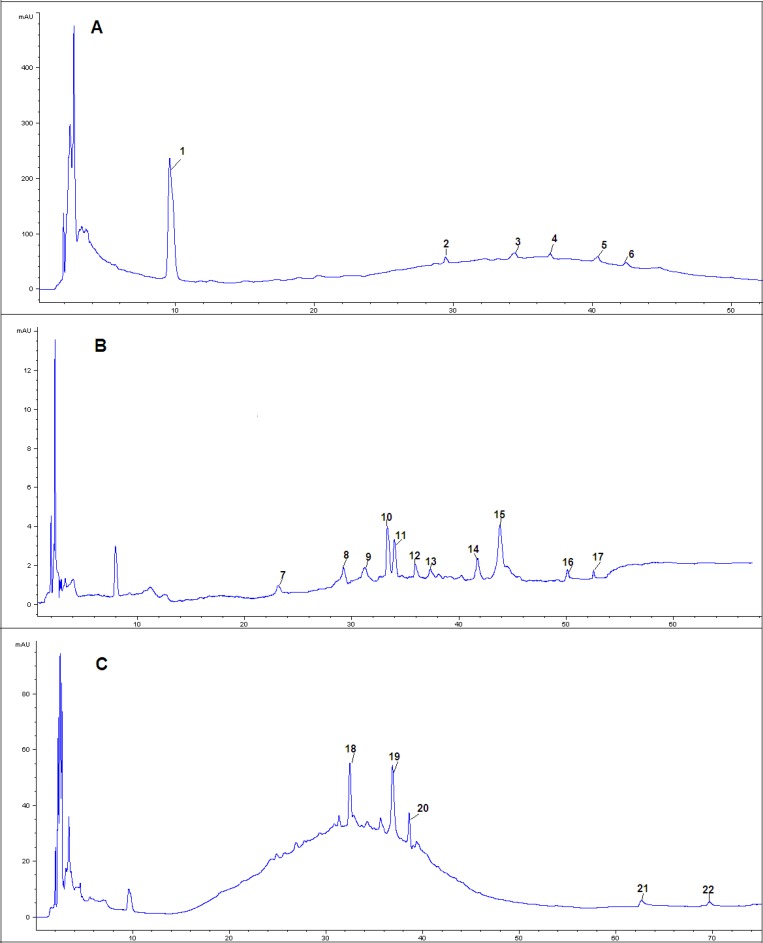
HPLC-PDA chromatograms of A(30% eluent), B(50% eluent) and C(70% eluent) of extract samples from *Ziziphus jujuba *Mill

*50% eluent*

Eleven compounds were found in 30% eluent. MS^2^ spectra of compound 7 (molecular ion at *m/z *[M-H]^-^ 193) gave characteristic fragment ions of [M-H-60]^-^ at *m/z *133. It corresponded to Quinic acid by comparison with literature data (17). MS^2 ^spectra of compound 8 (molecular ion at *m/z *[M-H]^-^ 671) gave characteristic fragment ions of [M-H-162]^-^ (lose of a hexose ) at *m/z *509, MS^2^ spectra of compound 10 (molecular ion at *m/z *[M+H]^+^ 439) gave characteristic fragment ions of [M+H-132]^+^ (lose of a pentose ) at *m/z *207, MS^2^ spectra of compound 12 (molecular ion at *m/z *[M+H]^+ ^453) gave characteristic fragment ions of [M+H-132]^+^ (lose of a pentose) at *m/z *321. Thus, Compound 8, 10 and 12 were all tentatively identified as Flavone glycoside. Compound 9 displayed a [M-H]^-^ ion at *m/z *609. Its MS^2^ fragmentation showed the loss of 308 Da (rutinose) resulting in a fragment ion at *m/z *301. Thus, compound 9 was characterized as Quercetin 3-O-rutinoside. MS^2^ spectra of compound 11 (molecular ion at *m/z *[M-H]^-^ 935) gave characteristic fragment ions of [M-H-162]^-^ (lose of a hexose ) at *m/z *773 and [M-H -162-162]^-^ (lose of two hexoses) at *m/z *611. Thus, compound 11 was presumed as O-triglycosyl saponin. Compound 13 gave a [M+H]^+^ ion at *m/z *579. MS^2^ fragmentations showed a loss of 278 Da (a rhamnose and a pentose) to form a fragment ion at *m/z *301. MS^3^ fragmentations of this ion gave a fragment ion at *m/z *245 due to the loss of 56 Da residue. Thus, compound 13 was tentatively indentified as Chrysoeriol-O-pentosylrhamnoside. Compound 14 gave a [M+H]^+^ ion at *m/z *1115, and the major product ions were [M+H-162]^+^ (*m/z *953), [M+H-162-162]^+^ (*m/z *791), [M+H-162-278]^+^ (*m/z *675), [M+H-162-278-162]^+^ (*m/z *513). The fragment of 278 Da corresponded to rhamnose and pentose. Three hexoses, one rhamnose and one pentose were detected in MS^2^ fragmentations. Compound 14 was tentatively indentified as O-polysaccharide glycosyl saponin. Compound 15 gave [M+Na]^+^ at *m/z *966 in MS spectrum, [M+Na-162]^+^ (lose of a hexose) at *m/z *804 and [M+Na-162-146]^+^ (lose of a hexose and a rhamnose) at 658 in MS^2^ spectra. However, it was not sure that the hexose was glucose or galactose for their same fragment (162 Da). Thus, compound 15 was characterized as Jujubasaponin ӀV or Jujubasaponin V. Compound 16 displayed a [M-H]^-^ ion at *m/z *959 and the major product ions were [M-H-18-162]^-^ (*m/z *779), [M-H-18-162-146]^-^ (*m/z *633) and [M-H-18-162-146-162]^-^ (*m/z *471). The fragment ions showed that there were two hexoses and one rhamnose in compound 16. However, it was not sure that the hexose was glucose or galactose for their same fragment (162 Da). Based on the mass spectral characteristics, compound 16 was tentatively identified as Lotoside Ӏ or Lotoside ӀӀ. Compound 17 gave a [M+H]^+^ ion at *m/z *301, it corresponded to Chryseoriol. 

*70% eluent*

Five compounds were found in 70% eluent. According to the reference compound, compound 21 and 22 corresponded to Jujuboside B and Oleanic acid, respectively. Compound 18 generated a [M-H]^-^ at m/z 911 in MS spectrum and a [M-H]^-^ at *m/z *749([M-H-162]^-^, lose of hexose), 603([M-H-162-146]^-^, lose of rhamnose) in MS^2^ spectra. However, it was not sure that the hexose was glucose or galactose for their same fragment (162 Da). Thus, compound 19 was characterized as Zizyphus saponin І or Zizyphus saponin ІІ. MS^2^ spectra of compound 19 (molecular ion at *m/z *[M+H]^+^ 683) gave fragment ions of [M+H-86]^+^ at *m/z *597 and [M+H-86-162]^+^ at *m/z *435. Thus, compound 18 was tentatively indentified as Flavone glycoside. MS^2^ spectra of compound 20 (molecular ion at *m/z *[M+H]^+^ 444) gave fragment ions of [M+H-18]^+ ^at *m/z *426 and [M+H-18-162]^+^ at *m/z *264, its tentative identification was also flavone glycoside. 

*Metabolic study in serum sample*

**Figure 3:**
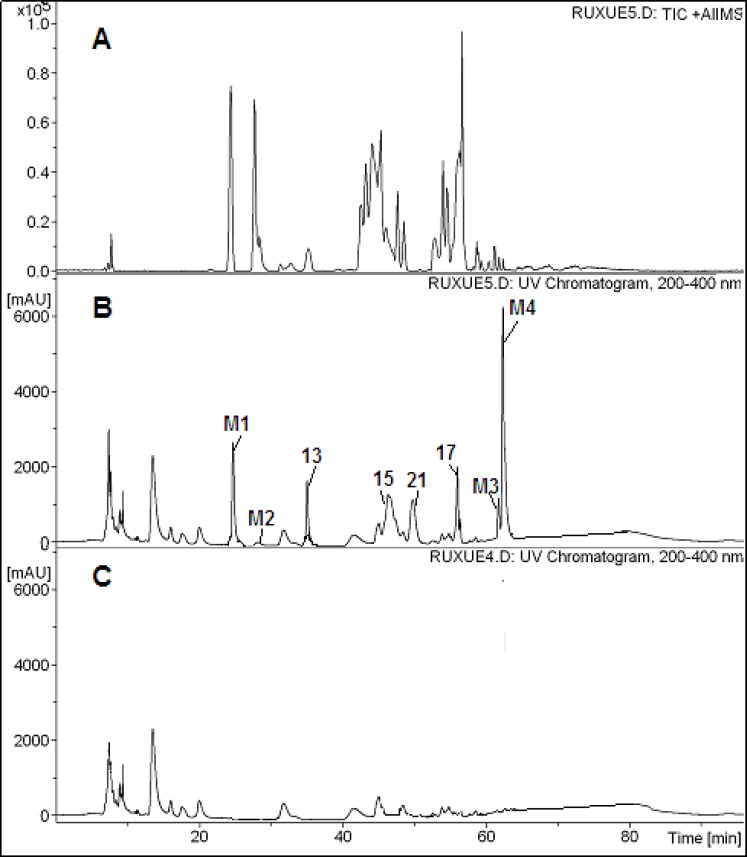
HPLC-PDA-ESI-MS^n^ chromatograms of serum samples after oral administration of the ethanol extract of Chinese jujube. A: Positive ion chromatogram of serum sample B: HPLC-PDA chromatograms of serum sample C: HPLC-PDA chromatogram of blank serum sample

The chromatograms obtained after by HPLC-PDA-ESI-MS^n^ analysis of serum samples after oral administration were shown in Figure 3 Compared with the chromatograms of the extract and the blank blood, four parent compounds, Chryseoriol, Chrysoeriol-O-pentosylrhamnoside, Jujubasaponin ІV (or Jujubasaponin V), Zizyphus saponin І (or Zizyphus saponin ӀӀ) were the main constituents in Chinese jujube, were detected in rat serum. In addition, four metabolites were also observed (Table 3). Metabolite 1 gaved a [M+H]^+^ ion at *m/z *796. MS^2^ spectra gave fragment ions of [M+H-60]^+^ at *m/z *736 and [M+H-60-124]^+^ at *m/z *612. The mass spectral characteristics of compound 2, 3 and 4 showed same fragmentation mechanism. Thus, they were presumed as metabolites. Further information would be required for the identification of the unknown compounds. 

**Table 3:** Peak assignments for rat serum of Ziziphus jujuba Mill

**No. **	**Identification **	**Molecular formula **	**UV, λ**_max_	**[M+H]** ^+^ **/ [M-H]**^-^	**MS/MS fragments **
13	Chrysoeriol-O-pentosylrhamnoside	C_27_H_30_O_14_	254	579/-	301, 245
15	Jujubasaponin ӀV orV	C_48_H_78_O_18_	203	966/-	804, 658
17	Chryseoriol	C_16_H_12_O_6_	254	301/-	
21	Jujuboside B	C_52_H_84_O_21 _	203	1067/-	935,789
M1	Unkown		278, 323	726/-	726, 666, 542
M2	Unkown		278, 323	796/-	736, 612
M3	Unkown		278, 323	820/-	760, 626
M4	Unkown		278, 323	836/-	776, 652

*Quantitative analysis of chinese jujube*

The contents of oleanolic acid, total sugars, total phenolics, flavones, flavonols, total saponins and cAMP were shown in Table 4. The content of oleanolic acid in ethanol extract was 0.070 ± 0.003 mg/g which was significantly higher than that in water extract, 0.036 ± 0.007 mg/g. In ethanol extract the contents of flavones and total saponins were also significantly larger than those in water extract. However, total sugars and cAMP contents of water extract were both higher than those of ethanol extract significantly. What’s more, statistical analyses showed that there was no significant difference in ethanol extract and water extract both total phenolics and flavonols. The result suggested that the effect of extraction solvents on contents of total phenolics and flavonols was little.

**Table 4:** Calibration curves data of quantitative analysis

**No.**	**Analyte**	**Methods**	**Reference compounds**	**Calibration surves ** a	**R** ^2^	**Linear range (mg/mL)**	**Contents of analytes (mg/g, n=3)**		
							**Ethanol extract**	**Water extract**
1	Oleanolic acid	HPLC	Oleanolic acid	*y*=5385.5*x*+5.984	0.9999	0.004~0.020	0.070 ± 0.003*	0.036 ± 0.007*	
2	cAMP	HPLC	cAMP	*y*=40605*x*-17.714	0.9998	0.002~0.050	0.049 ± 0.0013*	0.077 ± 0.0022*	
3	Polysaccharides	UV-Vis	Glucose	*y*=8.4893*x*-0.0049	0.9996	0.030~0.090	221 ± 11*	281 ± 18^*^	
4	Total phenolics	UV-Vis	Gallic acid	*y*= 6.785*x*-0.0213	0.9999	0.020~0.100	5.657 ± 0.09	5.407 ± 0.13	
5	Flavones	UV-Vis	Rutin	*y*=1.6567*x*+0.0024	0.9996	0.120~0.360	3.484 ± 0.25*	2.675 ± 0.31*	
6	Flavonols	UV-Vis	Quercetin	*y*=5.9733*x*-0.0387	0.9999	0.043~0.130	1.469 ± 0.05	1.425 ± 0.03	
7	Total saponins	UV-Vis	Jujuboside A	*y*=0.933*x*+0.0022	0.9998	0.100~0.500	9.501 ± 0.309*	7.750 ± 0.417*	

a *y *is the value of peak area (by HPLC method) and absorbance (by UV-Vis method), and *x *is the value of the reference compound’s concentration (mg/mL).

* Differences are significant at the 0.05 level.

## Conclusion

Twenty-two compounds were identified in the ethanol extract of *Ziziphus jujuba *Mill. The results implied that the Chinese jujube was rich in glycosides, flavanoids, nucleosides, organic acids and saponins. The method of HPLC-PDA-ESI-MS^n^ was simple and rapid for the identification of the flavonoids and saponins from Chinese jujube. Among them, seventeen compounds were reported for the first time. What’s more, D101 macroporous polystyrene resin was a good pretreatment method to obtain better separation and impurity removal effect. Four parent compounds (Chryseoriol, Chrysoeriol-O-pentosylrhamnoside, Jujubasaponin ІV or Jujubasaponin V, Jujuboside B) and four metabolites were detected in rat serum after oral administration of *Ziziphus jujuba *Mill. It meant they were the active constituent to play the pharmacological effect. The contents of oleanolic acid, total sugars, total phenolics, flavones, flavonols, total saponins and cAMP in Chinese jujube extracted by 70% ethanol and ultrapure water were investigated. 
